# Whole-Genome Sequence and Fermentation Characteristics of *Enterobacter hormaechei* UW0SKVC1: A Promising Candidate for Detoxification of Lignocellulosic Biomass Hydrolysates and Production of Value-Added Chemicals

**DOI:** 10.3390/bioengineering10091090

**Published:** 2023-09-16

**Authors:** Santosh Kumar, Eric Agyeman-Duah, Victor C. Ujor

**Affiliations:** Metabolic Engineering and Fermentation Science Group, Department of Food Science, University of Wisconsin-Madison, Babcock Hall, 1605 Linden Drive, Madison, WI 53706, USA; skumar232@wisc.edu (S.K.); agyemanduah@wisc.edu (E.A.-D.)

**Keywords:** *Enterobacter hormaechei*, whole-genome sequence, furfural, 5-hydroxymethylfurfural, lignocellulosic biomass, 2,3-butanediol, acetol

## Abstract

*Enterobacter hormaechei* is part of the *Enterobacter cloacae* complex (ECC), which is widespread in nature. It is a facultative Gram-negative bacterium of medical and industrial importance. We assessed the metabolic and genetic repertoires of a new *Enterobacter* isolate. Here, we report the whole-genome sequence of a furfural- and 5-hydroxymethyl furfural (HMF)-tolerant strain of *E. hormaechei* (UW0SKVC1), which uses glucose, glycerol, xylose, lactose and arabinose as sole carbon sources. This strain exhibits high tolerance to furfural (IC_50_ = 34.2 mM; ~3.3 g/L) relative to *Escherichia coli* DH5α (IC_50_ = 26.0 mM; ~2.5 g/L). Furfural and HMF are predominantly converted to their less-toxic alcohols. *E. hormaechei* UW0SKVC1 produces 2,3-butanediol, acetoin, and acetol, among other compounds of industrial importance. *E. hormaechei* UW0SKVC1 produces as high as ~42 g/L 2,3-butanediol on 60 g/L glucose or lactose. The assembled genome consists of a 4,833,490-bp chromosome, with a GC content of 55.35%. Annotation of the assembled genome revealed 4586 coding sequences and 4516 protein-coding genes (average length 937-bp) involved in central metabolism, energy generation, biodegradation of xenobiotic compounds, production of assorted organic compounds, and drug resistance. *E. hormaechei* UW0SKVC1 shows considerable promise as a biocatalyst and a genetic repository of genes whose protein products may be harnessed for the efficient bioconversion of lignocellulosic biomass, abundant glycerol and lactose-replete whey permeate to value-added chemicals.

## 1. Introduction

Global annual cellulosic biomass production is estimated to stand at 146 billion tons/year, which makes it the most abundant renewable resource on Earth [1]. Utilization of renewable lignocellulosic biomass materials (LBMs) as feedstocks to produce value-added chemicals that are currently derived from fossil fuels is a critical prerequisite to establishing a sustainable circular economy [2,3]. The efficient bioconversion of LBMs to bio-based chemicals hinges, to a great extent, on pretreatment and hydrolysis. However, during the release of sugars from LBMs during pretreatment, high temperatures in combination with the prevalent acidic/alkaline conditions co-generate inhibitory compounds, which currently represent the most challenging limiting factor impeding the takeoff of cellulosic chemicals [4,5]. Furfural, which is derived from the dehydration of pentose sugars during pretreatment, is undoubtedly the most abundant inhibitory compound released from LBMs [6,7]. In addition to furfural, the pretreatment of LBMs also generates an assortment of other inhibitory compounds at varying concentrations in lignocellulosic biomass hydrolysates (LBHs [8,9,10]), such as 5-hydroxymethylfurfural (HMF), 4-hydroxybenzaldehyde (4-HBD), *p*-coumaric acid, syringaldehyde, ferulic acid, vanillin, and cinnamaldehyde.

Furfural is inhibitory to a wide range of microorganisms [11]. For example, furfural concentrations above 1.0 mg/mL (~10 mM) inhibited growth and ethanol production by *Saccharomyces kluyveri* [12]. In a different study, 30 mM furfural significantly decreased H_2_ yield due to severe cell membrane damage in *Enterobacter aerogenes* [13]. According to Zhang et al. [14], ≤2 g/L furfural (~21 mM) and HMF (~16 mM) enhanced growth and butanol production by *Clostridium acetobutylicum*. However, when the concentrations of both inhibitors exceeded 2 g/L, cell growth was observed only after extended lag phases, with significant reductions in butanol yield. Accordingly, the degrees of growth inhibition and reduction in butanol yield increased significantly with increasing inhibitor concentration.

Several bacteria possess intrinsic tolerance to furfural and HMF, either via catalytic reduction to the less-toxic alcohols (transformation) or by utilization as carbon sources (mineralization). Many strains of *Pseudomonas putida* and *Cupriavidus basilensis* catabolize furfural into intermediates of the tricarboxylic acid (TCA) cycle through a series of largely oxidative reactions under aerobic conditions, using oxygen as the final electron acceptor [15,16,17]. In several other bacteria, furfural and HMF are reduced by NAD(P)H-dependent reductases and dehydrogenases to their corresponding less-toxic alcohols [7,8,9,18]. Consequently, the reduction route drains the cell of NAD(P)H, which is critical to biosynthetic reactions [18,19,20]. As a result, the catalytic reduction in the high concentrations of furfural and HMF in LBHs impairs central metabolism. In fact, a large number of microorganisms harbor the capability to catalytically reduce furfural and HMF to their less-toxic alcohols. However, the high concentrations of these inhibitors in LBHs place an enormous burden on cellular NAD(P)H pools, which elicits redox stresses that lead to the premature termination of fermentation.

To enhance furfural removal from LBHs, multiple strategies have been deployed. These include chemical removal, adaptive evolution, and strain engineering. In an effort to construct an inhibitor-detoxifying ‘juggernauts’, different authors have explored diverse strategies. For instance, cloning and expression of NADH-dependent benzyl alcohol dehydrogenases from *P. putida* and *Burkholderia ambifaria* in *E. coli* by Willson et al. [21] led to the rapid in vivo conversion of furfural to furfuryl alcohol, which in turn enhanced growth following furfural challenge. Cloning and expression of a native thymidylate synthase gene improved furfural resistance in *Escherichia coli* LY180 [20]. Furthermore, enhanced expression of *fucO, ucpA,* or *pntAB* and deletion of *yqhD* increased furfural tolerance in *E. coli* LY180 [22]. Similarly, chromosomal integration and expression of a native aldo-keto reductase gene (*akR*) in *Clostridium beijerinckii* NCIMB 8052 enhanced furfural, syringaldehyde and 4-HBD reduction, with a concomitant increase in butanol production [23]. Further, adaptive evolution using CRISPR-enabled trackable genome engineering (CREATE) improved furfural tolerance up to 4.7 g/L (~48.95 mM) in *E. coli* [24]. Indeed, the adroit mapping and characterization of well-studied organisms, as well as the isolation and characterization of novel strains and species with excellent inhibitor tolerance abilities—the basis of the present study—hold considerable promise for the efficient detoxification and fermentation of LBHs into renewable chemicals.

*Enterobacter* species are promising candidates for the efficient detoxification of LBH-derived inhibitors. In particular, several species of the ECC have been demonstrated to exhibit excellent lignocellulosic inhibitor detoxification and tolerance capabilities that may be harnessed for engineering more robust inhibitor-tolerant strains. For example, *Enterobacter cloacae* GGT036 was reported to tolerate a high concentration of furfural (IC_50_ = 47.7 mM; [25]). A survey conducted on the biotransformation of furfural and HMF by enteric bacteria, including an *Enterobacter* sp. in the presence of glucose and peptone, showed that enteric bacteria were able to convert furfural and HMF to their corresponding alcohols under both aerobic and anaerobic conditions in a relatively short incubation time (8 h; [26]). Furthermore, *Enterobacter* sp. FDS8 was shown to detoxify furfural and HMF at highly efficient rates of 0.54 g/L/h, and 0.12 g/L/h, respectively [27]. Thus, the high intrinsic tolerance to furfural and HMF and the ability to produce chemicals of commercial importance by *Enterobacter* species represents an attractive opportunity to overcome the current economic and technical hurdles associated with the bioconversion of LBMs to fuels and chemicals—either by the direct engineering of these organisms or by mining their genomes for target enzymes to be expressed in relevant organisms. In this study, we report the whole-genome sequence of *E. hormaechei* UW0SKVC1 (hereafter referred to as *E. hormaechei*), which exhibits high tolerance to furfural and HMF and grows on a wide range of sugars whilst producing a range of important biochemicals.

## 2. Material and Methods

### 2.1. Media and Culture Conditions

*E. hormaechei* was isolated as a contaminant on a Luria–Bertani (LB) agar plate (NaCl 10 g/L, tryptone 10 g/L, yeast extract 5 g/L, pH 7.0) while screening for furfural degradation by *P. putida*. *E. coli* DH5α was maintained and grown in LB media at 37 °C. Growth and furfural tolerance were measured at 37 °C in LB medium and in a mineral medium containing (per liter): glucose (10.0 g), Na_2_HPO_4_.2H_2_O (7.52 g), KH_2_PO_4_ (3.0 g), NaCl (0.5 g), NH_4_Cl (0.5 g), Na_2_MoO_4_.H_2_O (6 mg), H_3_BO_3_ (55 mg), CoCl_2_ (14 mg), CuSO_4_ (5 mg), MnCl_2_ (32 mg), ZnSO_4_ (6 mg), FeSO_4_ (2.5 mg), NiCl_2_, (2 mg) CaCl_2_.2H_2_O (14.7 mg), and MgSO_4_.7H_2_O (0.5 g). For fermentation and metabolite profiling using gas chromatography-mass spectrometry (GC-MS), cultures of *E. hormaechei* were grown in mineral medium containing glucose (60 g/L) or in rich medium [mineral medium supplemented with glucose (60 g/L), peptone and yeast extracts (5 g/L, each)]. Both media were adjusted to pH~7.5 before inoculation. Fermentations were conducted both aerobically and anaerobically. Anaerobic cultures were grown in 150 mL screw-capped bottles in an anaerobic chamber (Coy Laboratory Products Inc., Grass Lake, MI, USA) with a modified atmosphere of 82% N_2_, 15% CO_2_, and 3% H_2_ [28]. Aerobic cultures were inoculated with 1% (*v*/*v*) of the preculture and grown in a MAXQ 4450 rotary incubator (Thermo Fisher Scientific, Waltham, MA, USA) at 200 rpm. All cultures were grown in triplicate.

### 2.2. Assessment of Furfural and HMF Tolerance by E. hormaechei

The utilization of different substrates by *E. hormaechei* was tested in mineral media supplemented separately with D-glucose, D-xylose, D-arabinose or glycerol as a sole source of carbon. To assess furfural tolerance by *E. hormaechei*, increasing concentrations of furfural (0, 10, 30, 50 mM) were tested in LB broth supplemented with 1% (*w*/*v*) glucose. To assess the HMF tolerance capacity of *E. hormaechei*, 0, 10, 30, 50, and 70 mM HMF were supplemented in LB broth containing 1% (*w*/*v*) glucose. To determine if *E. hormaechei* uses HMF/furfural as a carbon source, *E. hormaechei* was grown on 20 mM furoic acid—an intermediate in the HMF/furfural catabolic pathway—which served as a sole carbon source. Additionally, *E. hormaechei* was grown on glucose (10 g/L) supplemented with 20 mM furoic acid. All cultures were grown in a MAXQ 4450 rotary incubator (Thermo Fisher Scientific, Waltham, MA, USA) at 37 °C and 200 rpm in 30 mL cultures. The optical densities of cultures were monitored every 2 h for 12 h at 600 nm using an Evolution 260 Bio UV-Visible spectrophotometer (Thermo Fisher Scientific, Waltham, MA, USA). The growth profiles of cells grown in the presence of furfural/HMF were compared to the control (without furfural/HMF).

### 2.3. Analytical Methods

High-performance liquid chromatography (HPLC) was used to quantify furfural, HMF, furfuryl alcohol and HMF alcohol concentrations in triplicate cultures of *E. hormaechei*. A total of 2 mL culture supernatant was collected every 2 h for 12 h, and then at 24 h. For HPLC analysis, samples and standards (1–10 mM) were diluted in methanol and then filtered using CellTreat (0.22 μm) syringe filters (CELLTREAT Scientific Products, MA, USA). The injection volume for each sample was 10 μL. HPLC analysis was carried out using an e2695 Waters HPLC system equipped with a 2998 PDA detector (Waters Corporation, Milford, MA, USA) and a Roc C18 column (Restek, Bellefonte, PA, USA) of 150 × 4.6 mm (length and internal diameter, respectively). The solvent was 50% (*v*/*v*) methanol in water and the flow rate was 1.0 mL/min. The UV detector wavelengths were set at 278 nm for furfural, 284 nm for HMF, and 218 nm for furfuryl alcohol and HMF alcohol. The column temperature was maintained at 40 °C. The mobile phase was filtered using Nalgene rapid flow 0.45 µm filters (Thermo Fisher Scientific, Waltham, MA, USA) connected to a pressure filtration unit (MasterFlex L/S Avantor, Radnor, PA, USA).

Analysis of the metabolites produced by *E. hormaechei* during fermentation was conducted by GC-MS. Cells were separately grown aerobically or anaerobically in mineral and rich media, as described earlier (under sample collection and growth conditions). During fermentation, 2 mL culture samples were collected at 0 h, 24 h, and 60 h time points, and then centrifuged at 16,000× *g* for 10 min in a Sorvall Legend Micro 21R centrifuge (Thermo Fisher Scientific, Waltham, MA, USA). Subsequently, 0.5 mL supernatant was drawn from each sample and freeze-dried overnight. Afterward, metabolites were extracted from the freeze-dried samples and analyzed by GC-MS according to the method previously described by Agyeman-Duah et al. [28].

To determine the concentrations of 2,3-butanediol (2,3-BD) produced by *E. hormaechei*, fermentations were performed in 150 mL flasks with 30 mL of the earlier described rich medium. Fermentations were conducted in triplicates for 24 h under anaerobic and aerobic conditions with either glucose or lactose (60 g/L) as the carbon source. Anaerobic fermentations were conducted in the anaerobic chamber, as described earlier [28]. Aerobic fermentations were spun at 200 rpm, and all cultures were grown at 35 °C. Samples were harvested after 24 h and the supernatant was collected by centrifuging the cells at 16,000× *g* for 10 min and 4 °C. The supernatants were diluted 10X in methanol and then analyzed by GC-MS using an Agilent 6890N gas chromatograph (Agilent Technologies Inc., Palo Alto, CA, USA) coupled to a mass selective detector (Agilent 5973 MS, Agilent Technologies Inc., Palo Alto, CA, USA), as previously described [28]. Standard concentrations of 2,3-BD (0.5, 1, 2, 4, 6, and 8 g/L) were analyzed by GC-MS to obtain a standard curve, which was used to quantify 2,3-BD concentrations in the culture supernatants of *E. hormaechei*.

### 2.4. DNA Extraction, Library Construction, and Whole-Genome Sequencing

Genomic DNA was extracted from *E. hormaechei* using the DNA extraction kit designed for high-molecular-weight DNA for long-read sequencing (Wizard^®^ HMW Promega, Madison, WI, USA). The quality of genomic DNA was assessed on 0.8% agarose gel and quantified using a Nanodrop (NanoDrop One; Thermo Fisher Scientific, Waltham, MA, USA). High-quality DNA (OD_260/280_ = 1.8–2.0) was used for library construction, as previously described [29]. Whole-genome sequencing (WGS) was conducted commercially by Novogene Inc. (Sacramento, CA, USA) using the Illumina platform (NovaSeq). The genomic DNA library for WGS was prepared by shearing DNA randomly into short fragments with an average length of 350 bp. The resultant fragments were end-repaired, A-tailed, and ligated with an Illumina adapter. These fragments were then amplified by PCR, size selected using a bioanalyzer, and then purified. Qubit and real-time PCR quantified libraries were pooled and sequenced on the Illumina platform (Illumina, San Diego, CA, USA).

### 2.5. Genome Assembly, Annotation and Gene Function Prediction

The accuracy and reliability of the raw sequencing data (1800 megabytes) were ensured by filtering out low-quality data as previously reported (i.e., reads with low-quality bases (mass value ≤ 20); [29,30]). The resulting clean data (1213 Mb, Q30 value = 94.11%) were assembled using the SOAP denovo software (version 2.04; [31,32]), SPAdes [33], and the ABySS assembling software [34]. Before assembly, the genome size was estimated by K-mer analysis [35]. The assembly results of the three software were integrated with the contig integrator for sequence assembly (CISA) software [36]. The gap-close software was used to fill the gaps in preliminary assembly results [37], and fragments < 500 bp were filtered out. The resulting data were used for gene and protein function prediction. Coding genes were predicted using GO, KEGG, COG, NR, and Pfam (blastp, evalue ≤ 1e^−5^) databases by adopting default parameters (identity ≥ 40%; coverage ≥ 40%). The prediction of non-coding RNAs, repetitive sequences, genomics islands, prophages, transposons, and clustered regularly interspaced short palindromic repeat sequences (CRISPR) was performed as previously described [38]. Various software, including GeneMarkS, were further applied to delineate coding genes [39]. tRNAscan-SE, rRNAmmer and BLAST were used against the Rfam database for noncoding RNAs (ncRNA; [40,41,42]), RepeatMasker was used for interspersed repeats [43], and Tandem Repeats Finder (TRF) was used for tandem repeat prediction [44].

### 2.6. Phylogenetic Analysis, Digital DNA-DNA Hybridization (DDH) and Average Nucleotide Identity (ANI) Analyses

For phylogenetic analysis, the complete genome sequence of *E. hormaechei* was uploaded to the type (strain) genome server (TYGS; https://tygs.dsmz.de (accessed on 15 November 2022)) for a whole-genome-based taxonomic analysis [45,46]. Two complementary methods were used to identify the type-strain genomes that showed the closest match to the user genome. First, using the modular arithmetic secure hash (MASH) algorithm, a quick approximation of intergenomic relatedness was conducted to compare the user genome to all type-strain genomes present in the TYGS database [47]. Subsequently, ten type strains with the least MASH distances to the user genome were selected. Second, the 16S rDNA gene sequences were used to identify an additional group of ten closely related type strains. Using RNAmmer, these were extracted from the user genomes [41] and each sequence was then BLAST-searched against the 16S rDNA gene sequence of each of the 17,385 currently accessible type strains on the TYGS database [48]. This was used as a proxy to find the best 50 matching type strains (according to the bit-score) for each user genome and to subsequently calculate precise distances using the genome blast distance phylogeny approach (GBDP) under the algorithm ‘coverage’ and distance formula d5 [49]. These distances were then used to determine the 10 closest type-strain genomes.

For the phylogenomic inference, all pairwise comparisons among the set of genomes were conducted using GBDP, and accurate intergenomic distances were inferred under the algorithm ‘trimming’ and distance formula d5 [49]. Digital DDH values and confidence intervals were calculated using the recommended settings of the genome-to-genome distance calculator (GGDC) 3.0 [46,49]. The resulting intergenomic distances were used to infer a balanced minimum evolution tree with branch support via FASTME 2.1.6.1, including SPR post-processing [50]. Branch support was inferred from 100 pseudo-bootstrap replicates. The trees were rooted at the midpoint [51] and visualized with PhyD3 [52]. The type-based species clustering using a 70% dDDH radius around each of the 14 type strains was carried out as previously described [45]. Subspecies clustering was conducted using a 79% dDDH threshold as previously described [53]. In addition, the level of pairwise genome-based similarities was evaluated based on the ANI value. The ANI used for species delineation (threshold below 94–96% indicates a new species) was determined by using the online OrthoANIu algorithm on the EzBioCloud server as described by Yoon et al. [54].

### 2.7. Genome Sequence Deposition and Data Availability

The WGS of *E. hormaechei* was deposited in the National Center for Biotechnology Information (NCBI) database using the Prokaryotic Genome Annotation Pipeline (PGAP version 6.0; [55]). The genome sequence submitted to the NCBI is assigned the BioProject accession number PRJNA884347, the BioSample accession number SAMN31019684, and the SRA accession number SRR22034406. This Whole Genome project has been deposited at DDBJ/ENA/GenBank under the accession JAPCKH000000000, and the version described in this paper is version JAPCKH000000000.1.

## 3. Results

### 3.1. Species Identification, Growth Profiles and Furan Tolerance of E. hormaechei

**Figure 1:**
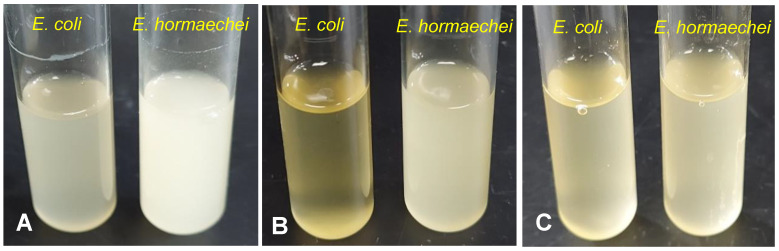
Growth profiles of *E. coli* DH5α and *E. hormaechaei* in LB medium supplemented with 1% (*w*/*v*) glucose and varying concentrations of furfural/HMF. (**A**) 0 mM furfural/HMF; (**B**) 30 mM furfural; (**C**) 40 mM HMF. Cultures were incubated for 24 h at 37 °C and 200 rpm.

*E. hormaechei* was identified based on ANI match details obtained from NCBI genome submission through automated Taxonomy-Check module, which showed 98.99% identity with type-strain *Enterobacter hormaechei* subsp. *Steigerwaltii.* Identification was further complemented by WGS-based phylogeny analysis, 16S rDNA sequence homology search, and the presence of a gene (*pqqB*)-encoding pyrroloquinoline quinone biosynthesis protein. The *pqqB* is a specific molecular marker encoding a small redox active molecule that differentiates *E. hormaechei* species from the genetically related *E. cloacae*. For the furfural and HMF tolerance study, gradient concentrations of furfural and HMF were added to a growth medium containing 1% (*w*/*v*) glucose. It was observed that *E. hormaechei* tolerates up to 30 mM furfural and 40 mM HMF (Figure 1A–C). The IC_50_ value of *E. hormaechei* for furfural was determined to be 34.2 mM, which is ~32% greater than the IC_50_ value calculated for *E. coli* DH5α (IC_50_ = 26.0 mM). However, the HMF tolerance of *E. hormaechei* (IC_50_ = 41 mM) was found to be comparable to that of *E. coli* DH5α. It was also observed that *E. hormaechei* grown in LB without the supplementation of 1% (*w*/*v*) glucose showed a 1.33-fold reduced tolerance to furfural (IC_50_ = 25.7 mM) when compared to cultures grown in LB medium with glucose (1%, *w*/*v*) supplementation. Additionally, when *E. hormaechei* was grown on 1% (*w*/*v*) glucose in the mineral medium, it exhibited much lower tolerance (1.9-fold) to furfural (IC_50_ = 18.0 mM), relative to cultures grown in the rich LB medium with 1% (*w*/*v*) glucose.

**Figure 2:**
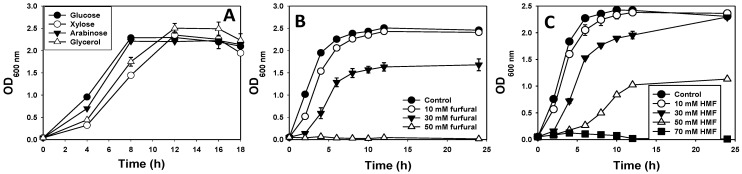
The growth profiles of *E. hormaechei* on different substrates, with and without furfural and HMF supplementation. (**A**) Growth on different carbon sources (glucose, xylose, arabinose and glycerol); (**B**) growth in LB medium supplemented with 1% (*w*/*v*) glucose and furfural (10 mM, 30 mM and 50 mM); (**C**) growth in LB medium supplemented with 1% (*w*/*v*) glucose and HMF (10 mM, 30 mM, 50 mM and 70 mM). The data presented here are the mean values ± SD calculated from three independent replicates.

The growth profiles on different carbon sources showed that *E. hormaechei* utilizes D-glucose, D-xylose, L-arabinose, and glycerol as sole carbon sources. Notably, in the first four hours, growth on xylose and glycerol lagged, relative to the growth profiles observed with glucose and arabinose (Figure 2A). However, at 12 h, the optical densities (OD_600 nm_) of xylose- and glycerol-grown cultures were identical to those observed with glucose and arabinose (Figure 2A). No growth was observed when furfural was used as a sole source of carbon. The growth of *E. hormaechei* was mostly inhibited in cultures containing ≥50 mM furfural. With 10 and 30 mM furfural, the growth rate (µ) of *E. hormaechei* reduced (µ = 1.71 ± 0.02 h^−1^ and µ = 1.16 ± 0.17 h^−1^, respectively) when compared to cultures not supplemented with furfural (µ = 1.82 ± 0.01 h^−1^; Figure 2B). Notably, the growth rate of *E. coli* DH5α was largely inhibited by 30 mM furfural (Figure 1B). Furthermore, in cultures supplemented with HMF, a decrease in growth rate was observed (µ = 1.73 ± 0.01 h^−1^ with 10 mM, µ = 1.17 ± 0.01 h^−1^ with 30 mM, and µ = 0.56 ± 0.01 h^−1^ with 50 mM) compared to the control cultures without HMF (µ = 1.8 ± 0.01 h^−1^). The growth of *E. hormaechei* was completely inhibited by 70 mM HMF (Figure 2C).

### 3.2. Furfural and HMF Are Largely Reduced to Their Less-Toxic Alcohols

**Figure 3:**
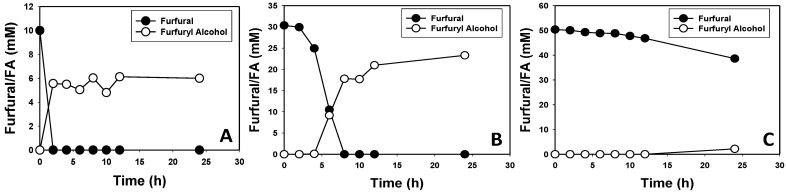
Furfural and furfuryl alcohol concentrations in furfural-supplemented cultures of *E. hormaechei.* Cells were grown in LB medium supplemented with 1% (*w*/*v*) glucose and different concentrations of furfural. (**A**) 10 mM furfural; (**B**) 30 mM furfural, and (**C**) 50 mM furfural. FA—furfuryl alcohol.

**Figure 4:**
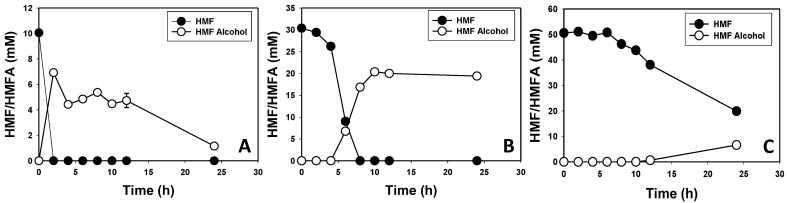
HMF and HMF alcohol concentrations in HMF-supplemented cultures of *E. hormaechei*. (**A**) 10 mM HMF; (**B**) 30 mM HMF, and (**C**) 50 mM HMF. HMFA—HMF alcohol.

HPLC analysis showed that *E. hormaechei* largely converts furfural and HMF to their less-toxic alcohols (furfuryl alcohol and HMF alcohol, respectively). When 10 and 30 mM furfural and HMF were supplemented into the culture medium, both furanic aldehydes were not detected in the culture supernatant after 2 h of growth for 10 mM and after 6 h for 30 mM furfural or HMF (Figure 3 and Figure 4). Cultures supplemented with 10 (~1.0 g/L), 30 (~2.9 g/L) and 50 (4.8 g/L) mM furfural transformed 100%, 100%, and ~42% of furfural to furfuryl alcohol at the rates of 0.48, 0.48, and 0.083 g/L/h, respectively (Figure 3). With 10 and 30 mM furfural, complete transformation was observed at 2 and 6 h, respectively (Figure 3A,B). Conversely, 58% furfural remained in the medium after 24 h in cultures supplemented with 50 mM furfural (Figure 3C). *E. hormaechei* exhibited greater tolerance to HMF than furfural. With 10 (1.26 g/L), 30 (3.8 g/L), and 50 (6.31 g/L) mM HMF, *E. hormaechei* transformed 100%, 100% and 60% of the HMF at the rates of 0.63, 0.47 and 0.16 g/L/h, respectively (Figure 4). Similar to furfural transformation, 10 and 30 mM HMF were completely transformed after 2 and 6 h, respectively, whereas only 60.2% HMF was transformed after 24 h in cultures supplemented with 50 mM HMF (Figure 4A–C).

It is noteworthy that although both furfural and HMF were mostly reduced to their respective alcohols, the amounts of furfuryl alcohol and HMF alcohol that accumulated in the respective cultures raised the question as to whether *E. hormaechei* utilizes both aldehydes as carbon sources, or at least converts a portion of both aldehydes to other intermediates other than their respective alcohols. For instance, with 10 and 30 mM furfural, 6 and 21.7 mM furfuryl alcohol were detected, respectively (Figure 3A,B). Thus, 40% and 27.7% of the transformed furfural was unaccounted for. Similarly, when cultures of *E. hormaechei* were challenged with 50 mM furfural, 20.9 mM was transformed, leaving behind 29.1 mM. However, only 5.6 mM furfuryl alcohol was detected in the culture. Consequently, 73.2% of the transformed furfural was unaccounted for. This trend was even more pronounced with HMF. Remarkably, 88.5%, 35.3% and 86.8% HMF were unaccounted for based on HMF alcohol concentrations in the cultures of *E. hormaechei* supplemented with 10, 30 and 50 mM HMF (Figure 4A–C). In fact, with 10 mM HMF (Figure 4A), ~7 mM HMF alcohol accumulated in the culture after 2 h. However, the concentration of HMF alcohol reduced progressively throughout the experiment such that, at 24 h, only 1.15 mM was present in the culture. Hence, between 2 h and 24 h, HMF alcohol concentration decreased by ~84%.

**Figure 5:**
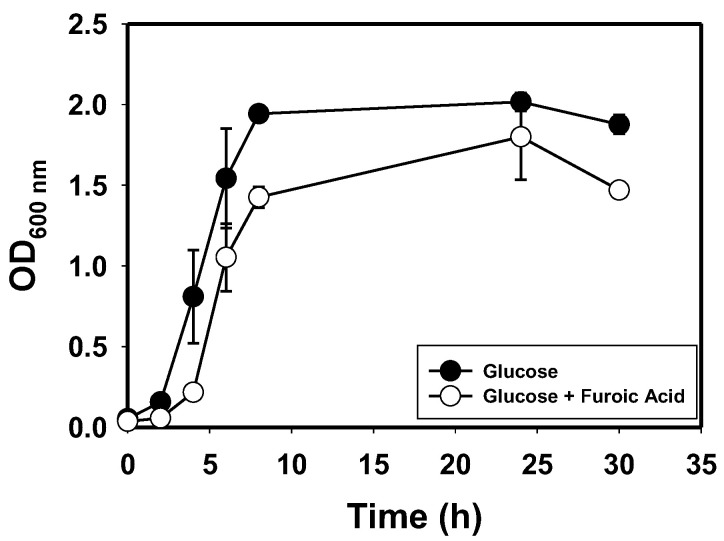
The growth profile of *E. hormaechei* in furoic acid supplemented glucose culture, relative to growth in the glucose control medium.

To further determine if *E. hormaechei* utilizes HMF/furfural as a carbon source, we grew this organism in cultures containing 20 mM furoic acid, with or without glucose (10 g/L) supplementation. With furoic acid alone as the sole carbon source, *E. hormaechei* did not grow. When furoic acid (20 mM) was added to the glucose (10 g/L) medium, growth was observed (Figure 5). However, in furoic acid-supplemented cultures, growth was slower, reaching a maximum optical density 12% lower than that observed with glucose alone. This indicates that furoic acid is toxic to *E. hormaechei*. More importantly, furoic acid concentration did not change over the course of *E. hormaechei* growth.

### 3.3. Select metabolites of importance produced by E. hormaechei UW0SKVC1

A qualitative analysis of the culture supernatant of *E. hormaechei* grown aerobically and anaerobically was performed by GC-MS. Among other compounds, *E. hormaechei* produced 2,3-butanediol (2,3-BD), acetoin, ethanol, hydroxyacetone (acetol), acetate, and L-lactate, under varying growth conditions (Table 1). Among these compounds, we quantified the amount of 2,3-BD produced by *E. hormaechei*. The results suggest that 2,3-BD is the predominant metabolite produced by this organism under aerobic conditions (Table 1) when grown on glucose or lactose. A nine-fold higher 2,3-BD titer (42.64 g/L) was obtained with glucose under aerobic conditions when compared to cultures grown anaerobically (4.6 g/L). A similar observation was made when we replaced glucose with lactose (Table 1).

### 3.4. The Genomic Components of E. hormaechei UW0SKVC1 and Gene Annotations Analysis

**Figure 6:**
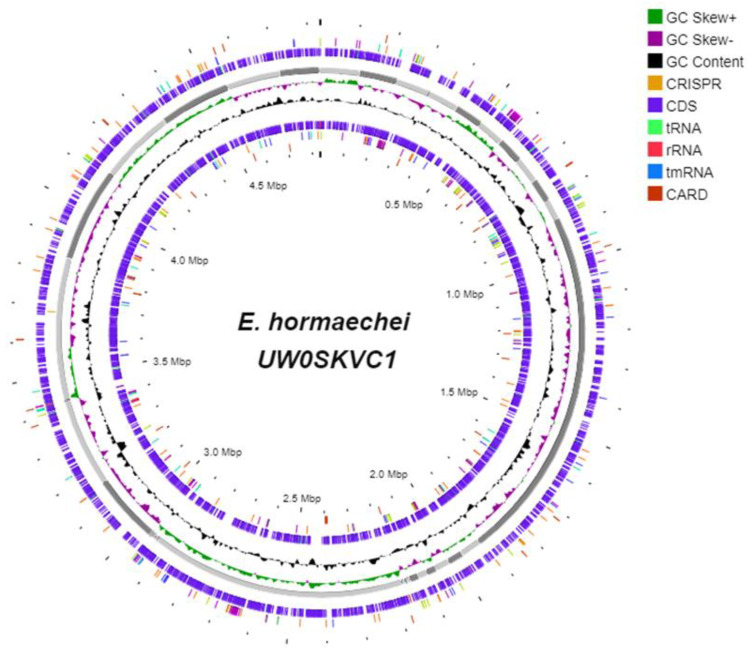
Circular map of the complete genome of *E. hormaechei* UW0SKVC1. Map was generated using the Proksee software system for genome assembly, annotation, and visualization (https://proksee.ca/ (accessed on 15 November 2022)). Detailed information on the genome assembly is available under GenBank accession numbers JAPCKH010000001.1 to JAPCKH010000047.1.

The genome sequence data of *E. hormaechei* revealed a total of 4,833,490 bp (N_50_ 278,784) assembled in 47 scaffolds with 55.35% G + C content. Homology-based gene prediction detected a total of 4580 protein-coding genes (88.78% of total genome; Figure 6 and Table 2). *E. hormaechei* contains a copious number of functional regions, which account for >90% of the genome.

The annotated genes were further divided into several subparts based on predicted functions (Table 3). The highest number of genes in *E. hormaechei* are related to carbohydrate metabolism, followed by genes encoding membrane transporters, which account for 7.54% and 7.29% of the total genes in this organism, respectively (Table 3).

### 3.5. Phylogenetic Trees, Digital DDH and ANI Analysis

**Figure 7:**
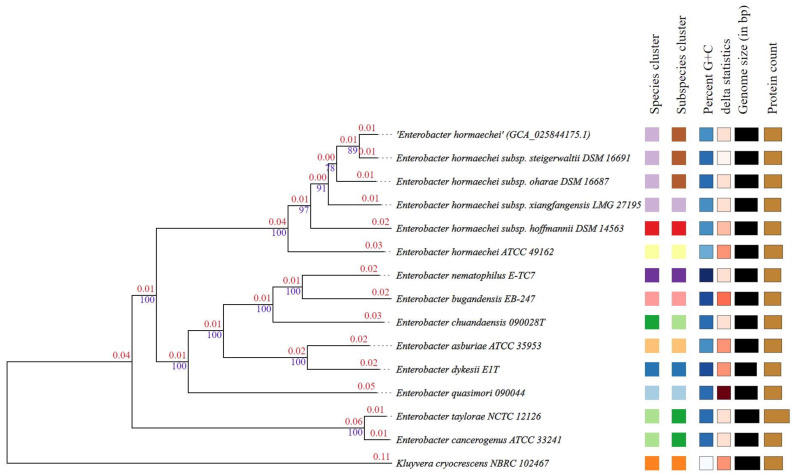
Phylogenetic tree constructed based on the whole genome of *E. hormaechei* UW0SKVC1. Tree was inferred with FastME 2.1.6.1 [50] from GBDP distances calculated from genome sequences. The branch lengths are scaled in terms of GBDP distance formula *d*_5_. The numbers above the branches are GBDP pseudo-bootstrap support values > 60% from 100 replications, with an average branch support of 96.5%. The tree was rooted at the midpoint. Color schemes (left to right) indicates the species and subspecies clusters based on similarities among strains. The same box colors indicate strains belonging to the same cluster as defined by each of the listed strains at the top of the boxes.

A phylogenetic tree based on the whole-genome sequence of *E. hormaechei* (phylogenomic analysis) was constructed using the type (strain) genome server (TYGS; https://tygs.dsmz.de/ (accessed on 15 November 2022)) database. The resulting species and subspecies clustering yielded 11 species and 12 subspecies clusters, and the provided sequence query strain was assigned to 1 of these clusters (shown by colored boxes in Figure 7). The resulting phylogenetic tree shows that *E. hormaechei* forms a distinct cluster composed of *E. hormaechei*-type species and that its close genetic neighbor is *E. hormaechei* subsp. *steigerwaltii* DSM 16691 (Figure 7). In-silico DNA-DNA hybridization values between *E. hormaechei* UW0SKVC1 and its close phylogenetic neighbors were calculated to confirm the taxonomic position of *E. hormaechei* UW0SKVC1, and the results suggest that strain UW0SKVC1 belongs to the *E. hormaechei*-type strain, as it was found to be well above the similarity threshold value of 70% recommended by Wayne et al. [56] to delineate prokaryotic genomic species (Table 4). Average nucleotide identity (ANI) analysis was further used to compare the similarity index between the genome of *C. hormaechei* UW0SKVC1 and other strains of the ECC that showed a close relationship to this strain. We determined that the ANI between *E. hormaechei* UW0SKVC1 and its close phylogenetic neighbors is 98.93% for *E. hormaechei* subsp. *steigerwaltii* DSM 16691, 97.74% for *E. hormaechei* subsp. *oharae* DSM 16687, 97.19% for *E. hormaechei* subsp. *xiangfangensis* LMG 27195, and 95.19% for *E. hormaechei* ATCC 49162—all of which are clearly above the 95-96% threshold used for prokaryotic species delineation [57,58]. Both the digital DDH and ANI values suggest that the strain described in this study is a strain of *E. hormaechei*, thus, we hereby assign the name *E. hormaechei* UW0SKVC1 to this strain.

### 3.6. Identification of Enzymes of Industrial and Environmental Important

**Figure 8:**
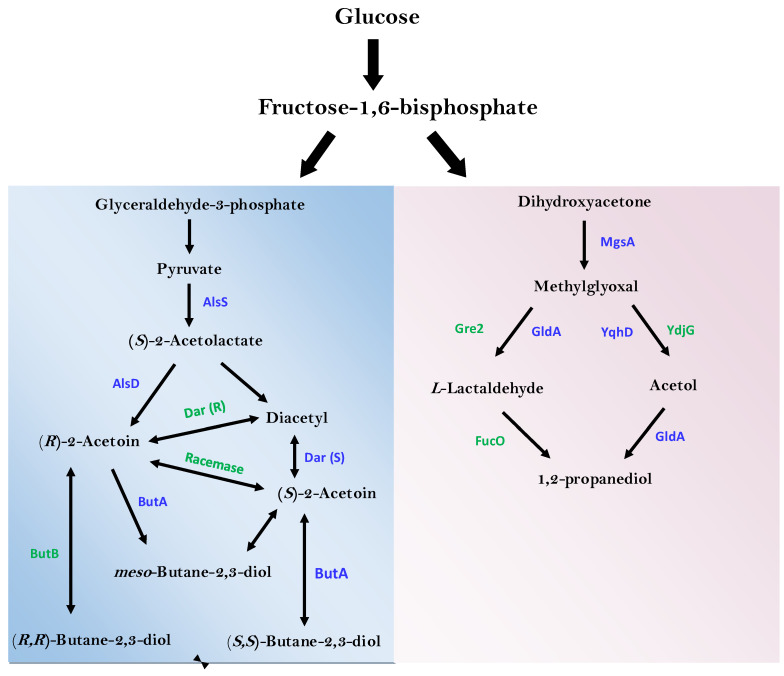
Availability of enzymes of the 2,3-BD (**left** light blue panel) and 1,2-PD (**right** grey panel) pathways in *E. hormaechei* UW0SKVC1. Enzymes in blue are present in the genome of *E. hormaechei*, whereas enzymes in green are absent.

A careful examination of the *E. hormaechei* genome relative to different metabolic pathways listed on the KEGG database showed that several genes encoding proteins involved in the degradation of xenobiotic compounds are present in this organism. Specifically, genes encoding enzymes involved in the degradation of aminobenzoate, naphthalene, nitrotoluene, xylene, and styrene were found to be present in the genome of *E. hormaechei*. Additionally, the genome of *E. hormaechei* also harbors genes of the 2,3-BD biosynthesis pathway (including genes that encode acetolactate synthase, acetolactate decarboxylase, and *meso*-butanediol dehydrogenase; Table 5 and Figure 8). Further, enzymes involved in propanoate metabolism, which is linked to the biosynthesis of 1,2-propanediol (1,2-PD), an industrially important bulk chemical, were also detected in the genome of *E. hormaechei*. To search for furfural degradation and detoxification-related enzymes in *E. hormaechei*, a protein blast was applied to align the amino acid sequences of this strain against the Swiss-Prot database. Remarkably, a high number of oxidoreductases and alcohol dehydrogenases that typically participate in biological oxidation and reduction in furfural and related aldehydes were found in *E. hormaechei*.

## 4. Discussion

Collectively, the inhibitory compounds, particularly the furans released during the pretreatment and hydrolysis of LBMs, represent a key limiting factor that hinders the use of renewable LBMs as feedstocks in the microbial production of value-added chemicals. Different organisms recruit a variety of mechanisms to combat these inhibitors during the fermentation of LBMs-derived sugars, with some exhibiting more robust inhibitor detoxification than others.

Thus, identification of microorganisms that exhibit strong tolerance to these furans holds promise for deploying metabolic engineering to construct robust strains that efficiently withstand LBMs-derived inhibitors during fermentation. Here, we report the whole-genome sequence of *E. hormaechei*, which tolerates 34.2 mM (~3.3 g/L) furfural and 40 mM (5 g/L) HMF. Previous reports showed that medium supplementation with 2 g/L furfural led to a 50% reduction in the growth of *E. coli* KO11, whereas the growth of *E. coli* LY01 was inhibited by up to 30% [59]. Conversely, as mentioned earlier, ≤2 g/L furfural and HMF stimulated the overall growth of *C. acetobutylicum*, albeit following a longer than normal lag phase [14]. However, concentrations of furfural and HMF greater than 2 g/L exert deleterious effects on solventogenic *Clostridium* species, leading to drastic reductions in growth and butanol production [60,61]. On the other hand, *S. cerevisiae* NCYC 3451 was reported to tolerate 3 g/L furfural [62], whereas Allen et al. [63] showed that strains of *S. cerevisiae* derived from strain FY10 tolerate 2.4 g/L (25 mM) furfural, following an extended lag phase. Interestingly, *Enterobacter cloacae* GGT036 tolerates ~3.4 g/L (35 mM) furfural [64], which is in agreement with the furfural tolerance ability observed in this study for the closely related *E. hormaechei* UW0SKVC1.

*Enterobacter* species tolerate comparatively higher concentrations of furanic aldehydes. This trend calls for greater attention, both for direct engineering to produce value-added chemicals and as sources of superior furan-detoxifying enzymes for heterologous expression in other species. Molecular tools for engineering *Enterobacter* species are available and continue to evolve rapidly, hence the need for additional efforts to harness their ability to tolerate higher concentrations of furanic aldehydes, moving towards establishing a bioeconomy. Boopathy et al. [26] demonstrated that a wide range of enteric bacteria (including *Klebsiella*, *Enterobacter*, *Escherichia*, *Citrobacter*, *Edwardsiella* and *Proteus*) exhibit strong tolerance to furfural and HMF, largely via transformation to their less-toxic alcohols. *Enterobacter* species are widely distributed in soil, in sewage, and in the gut—environments that have been shown to contain varying amounts of furfural, stemming from the degradation of plant materials [26,65]. Hence, the ability to tolerate furfural and HMF is thought to have originated from evolutionary adaptation following repeated exposure to furfural in soil, in sewage, and in the gut.

Furfural is formed during Maillard reaction in cooked food and has been reported in bread, popcorn, sterilized juice, meat, and sterilized alcoholic drinks [66,67]. Because *Enterobacter* species are prevalent in the gut, this might explain the preponderance of dehydrogenases and oxidoreductases in the genome of *E. hormaechei* UW0SKVC1, some of which certainly take part in the detoxification of furfural and HMF. The targeted identification of dehydrogenases and/or oxidoreductases that are abundantly expressed during the growth of *E. hormaechei* in furfural- and/or HMF-containing media will likely prove instructive. Apparently, *E. hormaechei* detoxifies both furfural and HMF largely via conversion to the respective alcohols. However, closer examinations of furfural, furfuryl alcohol, HMF, and HMF alcohol concentrations of furfural- and HMF-challenged cultures of this organism suggest that it might convert both aldehydes, particularly HMF (Figure 3 and Figure 4) to other metabolic intermediates. Typically, with organisms that only reduce furfural and HMF to their respective alcohols, approximately the same amounts of the alcohols (as the aldehydes: furfural and HMF) accumulate in the culture broth following aldehyde transformation. Remarkably, this is not the case with *E. hormaechei*. In fact, we observed ~30% to ~89% less furfuryl or HMF alcohol than the concentrations of furfural or HMF transformed during the growth of *E. hormaechei* (Figure 3 and Figure 4). Indeed, the concentration of HMF alcohol was reduced by ~84% between 2 h and 24 h (Figure 4A) in cultures supplemented with 10 mM HMF. 

This trend raised the question as to whether *E. hormaechei* might utilize a fraction of both or either aldehyde as a carbon source when they are co-supplied with glucose. However, furoic acid concentrations in the cultures grown on furoic acid as a sole carbon source or glucose-grown cultures supplemented with furoic acid firmly eliminated the possibility that *E. hormaechei* mineralizes HMF/furfural. Further, GC-MS analysis of the fermentation broths of furfural- and HMF-supplemented cultures did not detect any metabolic intermediate of furfural or HMF catabolism—particularly furoic acid. Furfural/HMF catabolism is characterized by a rapid reduction in their respective alcohols [15,16,67,68]. This eliminates the immediate toxicity of furfural or HMF. Subsequently, the alcohols are gradually absorbed and utilized as carbon sources via conversion to furoic acid and further to 2-oxoglutaric acid, which is then fed into the TCA cycle [15,67]. Taken together, we infer that *E. hormaechei* does not utilize HMF/furfural as a carbon source. Thus, the observed decreases in the concentrations of HMF alcohol and furfuryl alcohol cannot be accounted for by furfural or HMF catabolism. We will further investigate the fate of furfuryl and HMF alcohols in furfural- and HMF-supplemented cultures of *E. hormaechei* in future studies.

As with most *Enterobacter* species, the metabolic versatility of *E. hormaechei* further underscores its potential for likely industrial application. *E. hormaechei* utilizes glucose, xylose, and arabinose (Figure 2A)—all of which are derived from LBMs following deconstruction by pretreatment and hydrolysis. The ability to efficiently utilize lignocellulose-derived sugars is crucial to the economical deployment of LBMs within an industrial setting. Furthermore, this strain thrives on glycerol and lactose, both of which are abundant as waste residues from biodiesel production and cheese production, respectively. The genome of *E. hormaechei* contains the requisite genetic elements for the production of 2,3-BD, acetoin and 1,2-PD. Accordingly, 2,3-BD and its precursor (i. e., acetoin) were detected in cultures of *E. hormaechei* grown both aerobically and anaerobically. The compound 2,3-BD is a versatile industrial bulk chemical that finds application in food processing, synthetic rubber, lacquer and resin production, and as an anti-freeze, among several other applications [68,69,70,71]. Similarly, acetoin has sundry applications in food processing, cosmetics, agriculture and as an industrial solvent [71]. Our results show that 2,3-BD is the major product accumulated by *E. hormaechei*, particularly under aerobic conditions. Previous reports showed that the 2,3-BD titers of *Enterobacter* species range from 9.5 to 37.6 g/L [72,73,74,75]. Indeed, the strain of *E. hormaechei* described in this study is a potent producer of 2,3-BD. Furthermore, the genome of *E. hormaechei* contains the genes of the 1,2-PD pathway—a particularly important industrial bulk chemical used to make polyester resins and non-toxic de-icing agents [76].

The biological production of 1,2-PD has proven particularly challenging. Typically, 1,2-PD is produced via the 1,2-PD bypass, a poorly expressed glycolytic shunt that is thought to be deployed to dispose of excess sugar phosphates produced during high flux through glycolysis [76]. However, because this bypass involves the production of methylglyoxal, a highly toxic dicarbonyl compound [77], most potential 1,2-PD producers activate multiple mechanisms to circumvent the complete expression of the enzymes of this bypass, and in so doing, they produce little to no 1,2-PD. Although 1,2-PD was not detected in the cultures of *E. hormaechei*, its immediate precursor, hydroxyacetone (acetol), was present in the fermentation broth of this organism at different time points, both under aerobic and anaerobic conditions (Table 1). Hence, *E. hormaechei* holds promise as an engineering candidate for 1,2-PD production. The fact that acetol is produced by this organism suggests that tweaking the genetic repertoire of the 1,2-PD bypass in *E. hormaechei* might engender the biosynthesis of this important bulk chemical. Furthermore, acetol is an important industrial chemical used as a pharmaceutical intermediate and a food additive and is currently derived solely from petrochemicals [78]. Notably, using bioinformatics, we determined that the glycerol dehydrogenase gene (*gldA*), of which the protein product catalyzes the conversion of acetol to 1,2-PD, is present in the genome of *E. hormaechei* (Figure 8, Table 5). Therefore, the presence of acetol but not 1,2-PD in the culture of *E. hormaechei* suggests that *gldA* might be poorly expressed in this organism. Additionally, acetol might be secreted in trace amounts, which is likely not potent enough to elicit the expression of *gldA*. Given the challenges associated with the bio-production of 1,2-PD, establishing the expression profile of *gldA* in *E. hormaechei* will likely prove instructive.

In addition to the above-listed compounds, *E. hormaechei* also produces hexanoic and pentanoic acids (Table 1), both of which have significant industrial applications. For instance, hexanoic acid is deployed extensively in the manufacture of esters used in perfume production and in breweries, wine production and meat processing [79]. Similarly, pentanoic (valeric) acid is used extensively in the production of perfumes and cosmetics, as an additive in food processing, and as a plasticizer in pharmaceuticals [80]. The characterization of the hexanoic and pentanoic acid biosynthesis pathways in *E. hormaechei* will prove instructive towards engineering this organism for an improved accumulation of both acids.

In this study, we report the whole-genome sequence of *E. hormaechei* UW0SKVC1, which exhibits high tolerance to furfural and HMF. The presence of a nitrogen substrate and carbon source was found to be important for the efficient transformation of furfural and HMF to their respective alcohols. In addition to glucose, *E. hormaechei* UW0SKVC1 utilizes glycerol, lactose and pentose sugars (xylose, arabinose) that are commonly present in LBHs. Glycerol and pentose sugars are often less-favored carbon sources for many fermentative bacteria. Thus, *E. hormaechei* UW0SKVC1 holds considerable promise as a biocatalyst for direct furfural detoxification and the bioconversion of LBHs, glycerol and lactose-rich whey permeate to value-added chemicals such as acetoin, 1,2-PD or 2,3-BD. Further, the genome of this strain may offer new genetic targets for heterologous expression in more traditional fermentative workhorses, in the effort to valorize LBMs and other carbon sources for the production of biochemicals.

## Figures and Tables

**Table 1:** Select metabolites of importance produced by *E. hormaechei* UW0SKVC1.

Identified Compounds	* Mineral Medium				* Rich Medium			
	Aerobic		Anaerobic		Aerobic		Anaerobic	
	24 h	60 h	24 h	60 h	24 h	60 h	24 h	60 h
2,3-Butanediol	+	+	+	+	+	+	+	+
1,2-Cyclopentanedione	+	+	-	+	-	-	-	-
Acetoin (2-Butanone)	+	-	+	-	+	+	+	+
Ethanol	-	+	-	-	+	-	-	-
Acetic acid	+	+	+	+	+	+	+	+
Formic acid	-	-	-	+	+	-	+	+
Acetol (hydroxyacetone)	+	-	+	+	-	-	+	-
Hexanoic acid	+	+	+	+	-	-	-	-
Pentanoic acid	-	+	+	-	-	-	-	-
L-lactic acid	-	-	-	-	-	+	+	+
**2,3-BD concentrations (g/L)**								
	Aerobic				Anaerobic			
Glucose	42.64 ± 0.75				4.60 ± 0.2			
Lactose	41.65 ± 0.93				3.39 ± 0.097			

* Mineral medium: without yeast extract and peptone; Rich medium: mineral medium supplemented with yeast extract and peptone.

**Table 2:** Annotation statistics of functional genes, non-coding RNAs (ncRNA) and repeats in the genome of *E. hormaechei* UW0SKVC1.

Functional Annotation Against Databases (Protein Coding Genes)		Non-Redundant ncRNAs		Interspersed Repeats		Tandem Repeats	
nr KEGG SwissProt COG GO Pfam	4540 4508 3483 3845 3276 3276	tRNA 5 s (denovo) 16 s (denovo) 23 s (denovo) sRNA	78 8 1 1 41	LTR DNA LINE SINE RE Unknown	36 12 12 17 0 0	TR Minisatellite DNA Microsatellite DNA	78 52 1

LTR = long terminal repeat, LINE = long interspersed nuclear elements, SINE = short interspersed nuclear elements, RE = response element.

**Table 3:** List of biological pathways and annotated genes associated with the pathways in *E. hormaechei* UW0SKVC1.

Biological Pathway Type	No. of Genes	Biological Pathway Type	No. of Genes
**Cellular Processes** Transport and catabolism Cell growth and death Cell motility Cellular community—prokaryotes	8 177 69 22	**Metabolism** Xenobiotics biodegradation and metabolism Nucleotide metabolism Metabolism of terpenoids and polyketides Amino acids metabolism Metabolism of cofactors and vitamins Lipid metabolism Glycan biosynthesis and metabolism Energy metabolism Carbohydrate metabolism Biosynthesis of other secondary metabolites (e.g., siderophores)	37 105 33 298 182 66 57 164 340 36
**Environmental Information Processing** Signal transduction Membrane transport	169 329		
**Genetic Information Processing** Translation Transcription Replication and repair Folding, sorting and degradation	80 4 56 53		
**Human Diseases** Neurodegenerative diseases Infectious diseases Immune diseases Endocrine and metabolic diseases Drug resistance Cardiovascular diseases Cancers	6 40 2 6 72 7 18	**Organismal Systems** Nervous system Immune system Excretory system Environmental adaptation Endocrine system Digestive system Aging	2 4 1 8 12 2 9

**Table 4:** Calculated digital DDH values between *E. hormaechei* UW0SKVC1 and closely related type species of the genus *Enterobacter*.

Query Strain	Subject Strain	dDDH (d0, %)	C.I. (d0, %)	dDDH (d4, %)	C.I. (d4, %)	dDDH (d6, %)	C.I. (d6, %)	G + C Difference (%)
*E. hormaechei* UW0SKVC1	*Enterobacter hormaechei* subsp. *steigerwaltii* DSM 16691	87.3	[83.7–90.1]	91.4	[89.2–93.1]	90.7	[88.0–92.8]	0.19
	*Enterobacter hormaechei* subsp. *oharae* DSM 16687	85.1	[81.4–88.2]	80.6	[77.7–83.2]	87.3	[84.2–89.8]	0.22
	*Enterobacter hormaechei* subsp. *xiangfangensis* LMG 27195	81.8	[77.9–85.1]	76.1	[73.1–78.9]	83.7	[80.5–86.5]	0.08
	*Enterobacter hormaechei* subsp. *hoffmannii* DSM 14563	82.3	[78.4–85.6]	66.3	[63.3–69.1]	82.1	[78.8–85.0]	0.02
	*Enterobacter hormaechei* ATCC 49162	77.4	[73.4–80.9]	61.3	[58.4–64.1]	76.8	[73.3–79.9]	0.12
	*Enterobacter bugandensis* EB-247	71.8	[67.8–75.4]	35.5	[33.0–38.0]	62.7	[59.4–65.9]	0.64
	*Enterobacter quasimori* 090044	73.2	[69.3–76.9]	35	[32.6–37.5]	63.5	[60.2–66.8]	0.4
	*Enterobacter chuandaensis* 090028T	69	[65.1–72.7]	35	[32.5–37.5]	60.4	[57.1–63.6]	0.32
	*Enterobacter asburiae* ATCC 35953	61	[47.4–64.6]	34.6	[32.2–37.1]	54.3	[51.2–57.4]	0.11
	*Enterobacter chengduensis* WCHECl-C4	61.4	[57.7–64.9]	34.1	[31.7–36.6]	54.3	[51.2–57.4]	0.38
	*Enterobacter dykesii* E1T	67.2	[63.4–70.9]	33.8	[31.4–36.3]	58.5	[55.3–61.6]	0.49
	*Enterobacter taylorae* NCTC 12126	62.7	[59.0–66.3]	31.6	[29.2–34.1]	54.1	[50.9–57.2]	0.37
	*Enterobacter cancerogenus* ATCC 33241	66.1	[62.3–69.8]	31.4	[29.0–33.9]	56.3	[53.2–59.4]	0.32

Note: formula d0 (Genome-to-Genome Distance Calculator, GGDC, formula 1): length of all HSPs (heat shock protein) divided by total genome length, formula d4 (GGDC formula 2): sum of all identities found in HSPs divided by overall HSP length, formula d6 (GGDC formula 3): sum of all identities found in HSPs divided by total genome length. C.I. represents confidence intervals.

**Table 5:** Distribution of enzymes of the 2,3-BD and 1,2-PD pathways in *E. hormaechei* UW0SKVC1.

E.C. Number	Enzyme Symbol	Enzyme Name	Status in *E. hormaechei*
2.2.16	AlsS	Acetolactate synthase	Present
4.1.1.5	AlsD	Acetolactate decarboxylase	Present
1.1.1.303	Dar (R)	Diacetyl reductase	Absent
1.1.1.304	Dar (S)	Diacetyl reductase	Present
5.1.2.4		Acetoin racemase	Absent
1.1.1.4	ButB	(R,R)-Butanediol dehydrogenase	Absent
1.1.1.-	ButA	meso-Butanediol dehydrogenase	Present
1.1.1.76	ButA	(S,S)-Butanediol dehydrogenase	Present
4.2.3.3	MgsA	Methylglyoxal synthase	Present
1.1.-.-	YqhD	NADP-dependent alcohol dehydrogenase	Present
1.1.1.-	YdjG	Methylglyoxal reductase	Absent
1.1.1.6	GldA	Glycerol dehydrogenase	Present
1.1.1.283	Gre2	NADPH-dependent methylglyoxal reductase	Absent
1.1.1.77	FucO	Lactaldehyde reductase	Absent

## Data Availability

Data will be made available on request.
